# Lobar Collapse and Obliteration of Air Bronchogram Allowing Early Diagnosis of Endobronchial *Aspergillus* Infection following Hematopoietic Stem Cell Transplantation

**DOI:** 10.1155/2014/715073

**Published:** 2014-12-10

**Authors:** Elizabeth Thompson, Manigandan S. Thyagarajan, Elizabeth Johnson, David Weeden, Mary Morgan, Sarah J. Groves, Colin G. Steward

**Affiliations:** ^1^Faculty of Medicine and Dentistry, Senate House, Tyndall Avenue, Bristol BS8 1TH, UK; ^2^Department of Radiology, Birmingham Children's Hospital NHS Foundation Trust, Steelhouse Lane, Birmingham B4 6NH, UK; ^3^PHE Mycology Reference Laboratory, Public Health England Microbiology Services, South West Regional Laboratory Bristol Myrtle Road, Kingsdown, Bristol BS2 8EL, UK; ^4^Department of Thoracic Surgery, Southampton General Hospital, Tremona Road, Southampton SO16 6YD, UK; ^5^Department of Paediatric Haematology, Southampton General Hospital, Tremona Road, Southampton SO16 6YD, UK; ^6^School of Cellular & Molecular Medicine, Faculty of Medical Sciences, University of Bristol, University Walk, Bristol BS8 1TD, UK; ^7^Bone Marrow Transplant Unit, Royal Hospital for Children, Upper Maudlin Street, Bristol BS2 8BJ, UK

## Abstract

Endobronchial fungal infection (EBFI) is notoriously difficult to diagnose early since it may present few systemic features and does not cause characteristic parenchymal lesions on lung CT scanning. We report a 9-year-old girl who suffered extended neutropenia following graft failure after haematopoietic stem cell transplantation (HSCT) for severe aplastic anaemia. CT scan prior to retransplantation was normal despite persistent cough but lobar collapse was shown on repeat scan 16 days later. The probable diagnosis of EBFI (later proven on bronchoscopy) was only suspected when subsequent chest X-ray (CXR) demonstrated lack of an air bronchogram in the partially collapsed lung. Early radiological suspicion resulted in multiagent antifungal therapy followed by delayed lobectomy, and led to this being the first reported case of *Aspergillus* EBFI not to result in respiratory failure.

## 1. Introduction

Endobronchial fungal infection (EBFI) is a rare form of fungal invasion that is initially localized to the bronchial tree [1]. There are only three previous reports of endobronchial (EB) aspergillosis leading to lobar collapse with documented outcomes, all in patients with hematological malignancies [2–4]. In each case there was rapid decline to respiratory failure and two patients did not recover despite mechanical ventilation and antifungal therapy [2, 3]. We describe a patient with a benign hematological condition (severe aplastic anaemia, SAA) in whom serial chest X-rays (CXR) and CT scans showed progressive lobar collapse and loss of the air bronchogram. This led to clinical suspicion of EBFI, rapid escalation of antifungal therapy, confirmation of* Aspergillus fumigatus* infection through bronchoscopy, and definitive curative treatment with lobectomy. Early radiological investigations had been normal in this symptomatic patient. We discuss the value of repeat imaging and a high index of diagnostic suspicion in this underrecognized and underreported disease entity [1].

## 2. Case Report

A 9-year-old girl was admitted to the Bone Marrow Transplant Unit for a second haematopoietic stem cell transplantation (HSCT) procedure for SAA from her HLA-matched sister. Her blood count had returned to normal after her first transplant but she became pancytopenic again following a primary parvovirus infection 18 months after initial transplantation. This resulted in severe neutropenia for six months prior to admission whilst awaiting viral clearance. She was maintained on prophylactic cotrimoxazole and itraconazole throughout this period.

**Figure 1:**
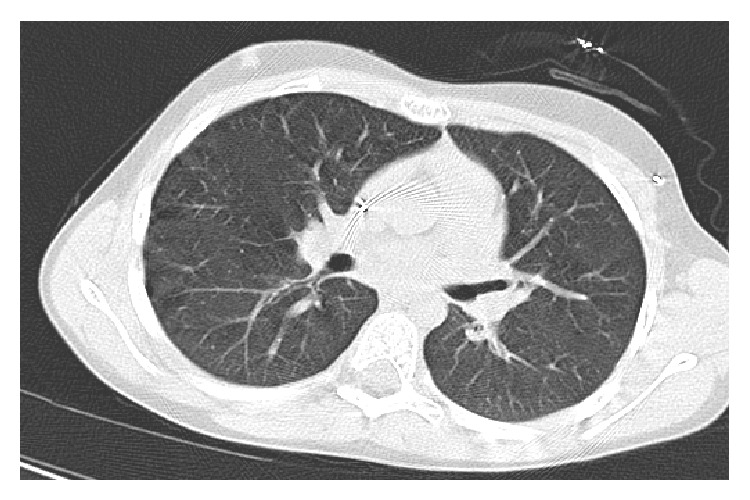
CT scan of the chest prior to start of HSCT. This showed no lung abnormality.

Prior to admission the patient had been nonspecifically unwell for a number of weeks, with a persistent cough, but was afebrile with a CRP of less than 10 mg/dL. Due to these symptoms she was thoroughly investigated on admission. Echocardiogram, lung function tests, and CT scan were all normal (the latter shown in Figure 1). The patient underwent one week of conditioning chemotherapy followed by HSCT and tolerated this well.

**Figure 2:**
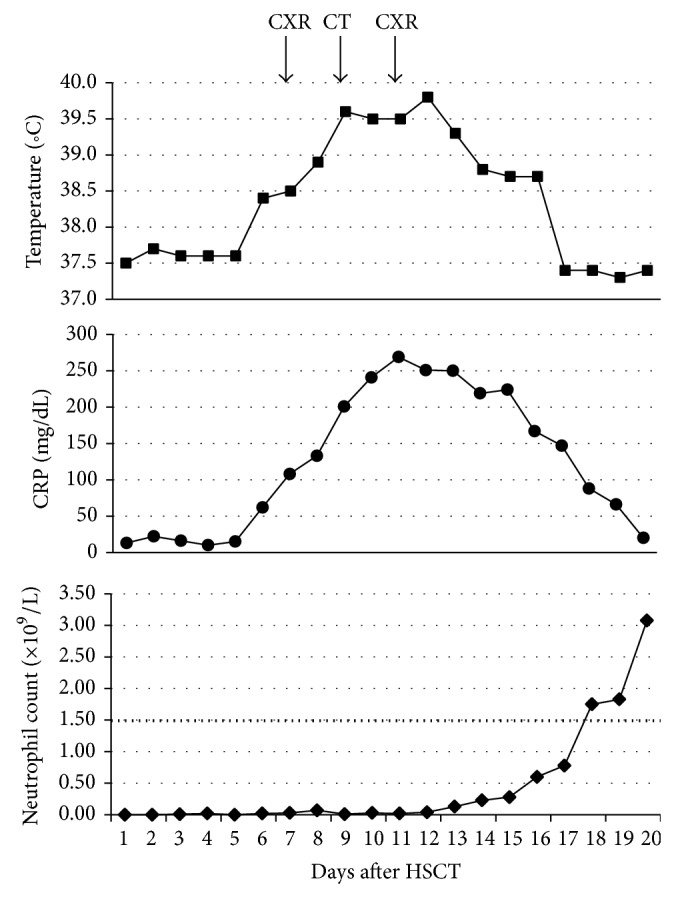
Graphs depicting temperature, CRP, and neutrophil count for 20 days following HSCT. Arrows indicate dates of CT and CXRs. The dotted line on the bottom panel indicates the threshold of neutropenia.

**Figure 3:**
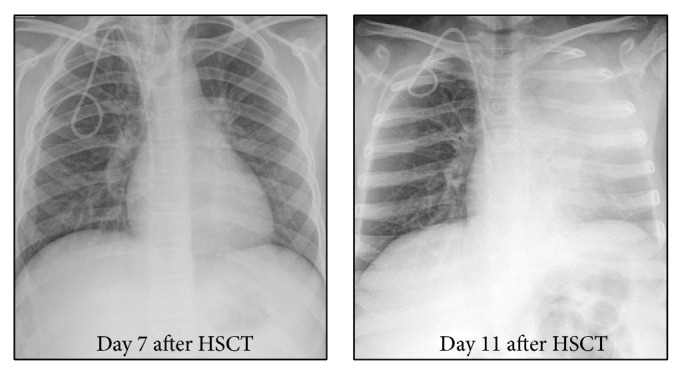
Chest X-rays performed on days 7 and 11 following transplant. No lung abnormality is seen at day 7 but left upper lobe collapse has developed by day 11 after HSCT.

**Figure 4:**
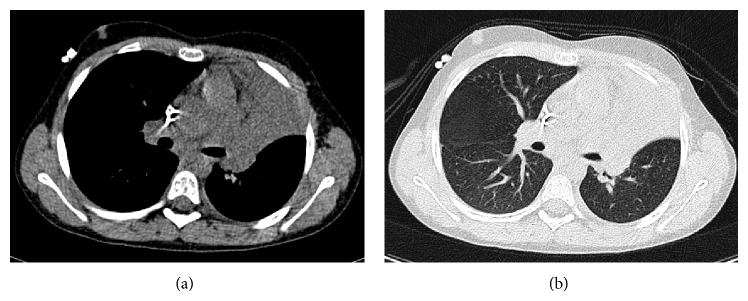
CT scan images of the chest performed on day 9 after transplant. (a) demonstrates complete collapse of the left upper lobe with abrupt cut-off of the left upper lobe bronchus and (b) absence of air bronchograms within the collapsed lung.

She remained well in herself (apart from her mild cough) until day 5 after transplant when she became febrile. Antibiotics were administered (piptazobactam, gentamicin, meropenem, and vancomycin in sequence according to local febrile neutropenia guidelines) but pyrexia worsened and CRP rose to 133 mg/dL on day 7 after transplant (clinical parameters shown in Figure 2). Chest radiograph (CXR) at this stage was clear (Figure 3). Daily intravenous liposomal amphotericin (1 mg/kg) was commenced in view of the previous protracted neutropenia. Additional symptoms of increasing tachypnoea and reduced breath sounds on examination prompted CT scanning on day 9 after transplant (Figure 4). This demonstrated left upper lobe collapse and a small left pleural effusion but no parenchymal nodules suggestive of fungal infection; nevertheless, caspofungin antifungal therapy was added due to the severity of the clinical situation.

Dyspnoea progressed and a CXR on day 11 after HSCT identified progressive lung collapse (Figure 3) with lack of an air bronchogram, noted to resemble foreign body obstruction, just proximal to the bronchus leading to the lingula lobe. In retrospect this could also be visualised on the CT image (Figure 4). An extrabronchial mass was deemed unlikely; therefore, an endobronchial mass was suspected, resulting in a high suspicion of an occluding EBFI. Although* Aspergillus* antigen testing at this time was negative, concern about clinical progression led to increased antifungal therapy: voriconazole (optimal first line therapy for invasive aspergillosis) was added to caspofungin and an increased dose of liposomal amphotericin (5 mg/kg) was used.

The patient initially exhibited a good response to triple antifungal therapy (pyrexia and dyspnoea resolved), supported by a rising neutrophil count (Figure 2). However, on day 19, she developed shoulder tip pain and by day 21 redeveloped a fever. Following exclusion of the more common causes of febrile illness seen in patients recovering their neutrophil count after HSCT, bronchoscopy was performed on day 27. This revealed a white plaque completely occluding the left main bronchus; slight suction on this mass produced profuse bleeding and bronchoscopy was aborted. Microscopy of bronchoscopic samples identified fungal hyphae and* Aspergillus fumigatus* was grown in culture. The patient remained afebrile but with a persistent CRP between 20 and 40 and was therefore commenced on additional nebulized amphotericin (5 mg BD, preceded by nebulised salbutamol) on day 38. Her CRP fell to 12 on this regime by day 48.

Whilst left lower lobe atelectasis resolved, the upper lobe collapse remained and lobectomy was required subsequently. This entailed a complex, eight-hour procedure which involved extensive bronchovascular dissection because of adherence of nodes to the hilar structures including opening of the pericardium and the left main bronchus. The patient remained well and free of* Aspergillus* infection five years later.

## 3. Discussion

EBFI predominantly affects severely immunocompromised patients such as those undergoing solid organ transplantation or HSCT or receiving cytotoxic chemotherapy [1, 5, 6]. This report focuses on* Aspergillus* infection which is the leading cause of EBFI, accounting for 53% of cases in a 238-case review [1]. This presentation has also been reported in coccidioidomycosis, mucormycosis, candidosis, cryptococcosis and histoplasmosis in descending order of frequency [1].

The potential for EB aspergillosis to progress rapidly to respiratory failure is depicted by previously reported cases [2–4]. Furthermore, requirement for mechanical ventilation drastically affects mortality rates, 94% compared to 25% in nonventilated populations [5]. This emphasises the importance of early diagnosis which, due to a lack of disease defining symptoms, invariably places heavy reliance upon investigations [5].

In EBFI the classic “halo” and “crescent” signs of invasive parenchymal aspergillosis will not show on CT scans due to the intraluminal nature of the infection (unless there is coexistent parenchymal disease) [1]. However, there may be more subtle suggestive signs including distal bronchiectasis, bronchial wall thickening, and, at a later stage, lobar collapse [1, 5, 6]. Some authors have suggested a limited role for CXR in the detection of these infections and recommend instead the use of high-resolution CT scanning [5, 6]. In the patient described both lung CT and CXR initially failed to identify abnormalities when cough and weight loss were the only symptoms. Although lobar collapse was later detected by both imaging modalities, the underlying diagnosis was only suspected when review of a subsequent CXR revealed bronchial obstruction with loss of an air bronchogram in the progressively collapsing lung. This triggered CT scan review which retrospectively identified the point of bronchial obstruction.

Anecdotally, during our discussions of this case with members of the HSCT community, we have found that many senior transplant professionals are unaware of the existence of occlusive EBFI and its difficulties of detection on CT scanning. This is crucial when CT has become the gold standard investigation for fungal infection in HSCT patients. Bronchoscopy is now much less widely used than previously in HSCT patients because of the advent of PCR viral detection systems for throat swabs/sputum analysis and because of the ease and efficacy of high-resolution CT scanning. This case demonstrates that it is still crucial to consider bronchoscopy if these investigations are negative but the patient is persistently febrile and/or develops respiratory signs/symptoms.

This report illustrates the nonspecific clinical presentation of EBFI and therefore the high index of suspicion required to diagnose these patients. Radiological suspicion played a pivotal role in rapid escalation of antifungal therapy; it was almost certainly this which prevented progression to respiratory failure and ventilation and enabled a curative outcome for this patient [5].
